# Dexamethasone as Adjuvant to Bupivacaine Prolongs the Duration of Thermal Antinociception and Prevents Bupivacaine-Induced Rebound Hyperalgesia via Regional Mechanism in a Mouse Sciatic Nerve Block Model

**DOI:** 10.1371/journal.pone.0123459

**Published:** 2015-04-09

**Authors:** Ke An, Nabil M. Elkassabany, Jiabin Liu

**Affiliations:** 1 Department of Anesthesiology & Critical Care, Perelman School of Medicine, the University of Pennsylvania, Philadelphia, Pennsylvania, United States of America; 2 Department of Anesthesiology, The first affiliated hospital of Sun Yat-Sen University, Guangzhou, P.R. China; University of Texas Medical Branch, UNITED STATES

## Abstract

**Background:**

Dexamethasone has been studied as an effective adjuvant to prolong the analgesia duration of local anesthetics in peripheral nerve block. However, the route of action for dexamethasone and its potential neurotoxicity are still unclear.

**Methods:**

A mouse sciatic nerve block model was used. The sciatic nerve was injected with 60ul of combinations of various medications, including dexamethasone and/or bupivacaine. Neurobehavioral changes were observed for 2 days prior to injection, and then continuously for up to 7 days after injection. In addition, the sciatic nerves were harvested at either 2 days or 7 days after injection. Toluidine blue dyeing and immunohistochemistry test were performed to study the short-term and long-term histopathological changes of the sciatic nerves. There were six study groups: normal saline control, bupivacaine (10mg/kg) only, dexamethasone (0.5mg/kg) only, bupivacaine (10mg/kg) combined with low-dose (0.14mg/kg) dexamethasone, bupivacaine (10mg/kg) combined with high-dose (0.5mg/kg) dexamethasone, and bupivacaine (10mg/kg) combined with intramuscular dexamethasone (0.5mg/kg).

**Results:**

High-dose perineural dexamethasone, but not systemic dexamethasone, combined with bupivacaine prolonged the duration of both sensory and motor block of mouse sciatic nerve. There was no significant difference on the onset time of the sciatic nerve block. There was “rebound hyperalgesia” to thermal stimulus after the resolution of plain bupivacaine sciatic nerve block. Interestingly, both low and high dose perineural dexamethasone prevented bupivacaine-induced hyperalgesia. There was an early phase of axon degeneration and Schwann cell response as represented by S-100 expression as well as the percentage of demyelinated axon and nucleus in the plain bupivacaine group compared with the bupivacaine plus dexamethasone groups on post-injection day 2, which resolved on post-injection day 7. Furthermore, we demonstrated that perineural dexamethasone, but not systemic dexamethasone, could prevent axon degeneration and demyelination. There was no significant caspase-dependent apoptosis process in the mouse sciatic nerve among all study groups during our study period.

**Conclusions:**

Perineural, not systemic, dexamethasone added to a clinical concentration of bupivacaine may not only prolong the duration of sensory and motor blockade of sciatic nerve, but also prevent the bupivacaine-induced reversible neurotoxicity and short-term “rebound hyperalgesia” after the resolution of nerve block.

## Introduction

Regional anesthesia is gaining popularity with the potential benefits of improved analgesia, reduced nausea and/or vomiting, and improved patient satisfaction [1]. Peripheral nerve blocks (PNBs) have been delivered via either single injection or continuous catheter infusion approaches [2]. The single injection nerve block is easier to perform and requires less resources in follow up management, which is more cost-effective in our clinical practice [3]. However, solitary nerve block is limited by the duration of effective analgesia coverage. The available choice of local anesthetics (LA) and maximum toxic dosage preclude the amount of local anesthetic that can be used with single injection. Finding adjuvants to the local anesthetic that could effectively and reliably extend the analgesia duration has been the focus of researchers’ efforts recently [4].

One promising adjuvant is dexamethasone. Dexamethasone is a synthetic glucocorticoid drug with potent anti-inflammatory and immunosuppressant effects. Several studies have reported that 8–10 mg of perineural dexamethasone can significantly prolong the analgesia duration of brachial plexus nerve block [5–9]. Two recent publications reported comparable effects on prolongation of analgesia duration between perineural and intravenously administered dexamethasone [10, 11], which lead to the assumption that the mechanism of dexamethasone as adjuvant in peripheral nerve block might be systemic in nature.

In previous studies, the dosage of dexamethasone used in peripheral nerve block was simply determined based on intravenous equivalent dose. Even though the mg per kg body weight dosage is reasonable, the local concentration as well as the amount of dexamethasone locally is many fold higher. The potential neurotoxicity of dexamethasone to peripheral nerve is still a serious concern awaiting research clarification. This study aims to answer the following questions: what is the appropriate and effective dosage for dexamethasone as adjuvant in peripheral nerve block? Is there any neurotoxicity associated with dexamethasone? What is the potential mechanism of dexamethasone-induced analgesia prolongation observed in clinical studies?

## Materials and Methods

### Ethics Statement

The study was approved by the Institutional Animal Care and Use Committee (IACUC) at University Laboratory Animal Resources of the University of Pennsylvania (Philadelphia, PA, USA. Protocol# 803980). The study followed the Use of Laboratory Animals and the Guide for the Care and Use of Laboratory Animals (1996).

### Drug preparation

Commercially available 0.75% bupivacaine (APP Pharmaceuticals, LLC, Schaumburg, IL, USA.) was mixed with preservative-free normal saline or dexamethasone sodium phosphate (10 mg/mL, preservative-free; APP Pharmaceuticals, Schaumburg, IL, USA.). The final bupivacaine concentration was 0.5%, and the pH for all medication preparations were maintained at 6.00±0.48.

### Study groups

There were six study groups. Three control groups included normal saline, 10mg/kg bupivacaine, and 0.5mg/kg perineural dexamethasone. The three experimental groups included 10mg/kg bupivacaine with 0.14mg/kg perineural dexamethasone, 10mg/kg bupivacaine with 0.5mg/kg perineural dexamethasone, and 10mg/kg bupivacaine with 0.5mg/kg intramuscular dexamethasone.

### Animals and paw withdrawal latency testing

Male 129S retired breeder mice (n = 60, weight 28–30g) were purchased from Charles River Laboratories (Wilmington, MA, USA.). All mice were housed at room temperature (20–25°C) under a 12–12h light-dark cycle with free access to food and water *ad libitum*. Thermal hyperalgesia thresholds were measured as previously described in the literature [12]. Mice were placed on a preheated glass platform within a plastic chamber. After the mouse was habituated to the plexiglass chamber in a Hargreaves apparatus (Model 37370, Ugo-Basile, Comerio, Italy), the plantar surface of affected hind paw was exposed to a beam of radiant heat through a transparent perspex surface. Paw withdrawal latency (PWL) to the radiant heat stimulus was recorded with the infrared intensity set at 50%, cut-off time 15s, and reaction time 0.1s. The heat stimulation was repeated 3 times at an interval of 2–3 min for each paw and the mean values were then calculated. All mice were conditioned to the paw withdrawal chambers for 1h per day and PWL to heat stimulation values were obtained on both the operative and control paws 2 days before surgery. The mean value of five measures was recorded as the baseline value.

### Perineural sciatic nerve and intramuscular injection

Male 129S mice were anesthetized and maintained with 1–3% isoflurane (Piramal Critical Care Inc., Bethlehem, PA, USA) in oxygen. Upon achieving sufficient depth of anesthesia, mice were placed in the prone position. A cut-down incision of 0.5–1 centimeter was made at in the upper thigh. Under minimal dissection, the sciatic nerve was identified in the intermuscular interval between the biceps femoris and gluteal muscle without dissection of superficial fascia layers. Then, a mixture of bupivacaine with or without dexamethasone (60μL) was injected into the perineural space below the fascia using a tuberculin syringe with a 30-gauge needle. In order to determine the effect of systemic administration of dexamethasone on the bupivacaine block, dexamethasone (0.5mg/kg) was injected intramuscularly in the systemic dexamethasone group right before sciatic nerve injection with bupivacaine. The time of the injection was recorded and deemed as the zero time point. The surgical wounds were closed with stainless steel wound clip (Reflex 7, Cellpoint Scientific INC., Gaithersburg, MD, USA).

### Neurobehavioral examination

Upon completion of the surgery, the anesthetized mice were returned to their cages and placed in the supine position. After righting, mice were placed in the chamber for PWL testing. Three PWL measures from both the operative and the control paws were obtained every 15–30 min for the first two hours from the time of the injection, then every hour until the return of normal paw withdrawal response. The mean value of the three measurements at each time point was calculated. In addition, the motor function of the surgical hind paw was also assessed every 5 min by observation as either a curled paw (motor score = 1, indicates motor blockade) or a normal paw position (motor score = 0, no motor blockade) [13]. PWL test was repeated every day after surgery up to day 7. For mice scheduled for nerve harvest on day 2 or day 7, additional six PWL measurements were taken immediately before euthanasia and nerve harvest.

### Tissue harvesting

Previous studies have indicated that early structural changes associated with axonal degeneration occur between 2 and 7 days after injury [14]. All mice were anesthetized as described above, and then approximately 8–10 millimeters of sciatic nerve from the sciatic notch to the mid-thigh were gently exposed and removed. The sciatic nerves were immediately immersed in 4% paraformaldehyde in 0.1M phosphate buffer (Alfa Aesar, Johnson Matthey Company, Ward Hill, MA, USA) at 4°C in fixative for 24–72hrs, then washed three times and transferred to a phosphate buffer for further testing.

### Histopathological and immunohistochemical evaluation of sciatic nerve

Previous studies showed that abnormal axon and myelin degeneration could be profiled as myelin figures, which have both the myelin and axoplasm combined in a dark-staining mass suggestive of more advanced nerve degeneration [15]. The sections were put on an inverted microscope (Olympus IX3 Phase; Olympus Optical Co, Ltd, Tokyo, Japan) and the degeneration profiles were identified by light microscopy and expressed as a percentage of demyelinated axon in each total field of vision (200× magnification) and quantified with ImageJ 1.49a software (NIH, Bethesda, Maryland, USA). Similarly, the nucleus percentage and protein immunoreactivity (average optical density, AOD) in each nerve section were measured. Ten nerves in each group (five at 2 days and five at 7 days) were analyzed. They were dehydrated in graded concentrations of ethanol, and embedded in paraffin. Cross-sections of sciatic nerve, 3–5μm thick, were stained with 0.1% toluidine blue (Arlington, TX, USA), cleaved Caspase-3 (Asp175) (5A1E) rabbit mAb (1:400, Cell Signaling Technology, Danvers, MA, USA), S-100 protein rabbit polyclonal antibody (1:200, Vector Laboratories, Inc., Burlingame, CA, USA), and counterstained with hematoxylin for nuclei (Fisher Scientific, Pittsburgh, PA, USA). All sample processing and interpretation were conducted through fee-for-service at Cancer Histology Core of Abramson Cancer Center at the University of Pennsylvania. Toluidine blue staining, caspase-3 staining, and S-100 expression were interpreted according to service core standard. Additional positive control for caspase-3 staining was conducted on mouse hippocampus to confirm the actual low incidence of positivity.

### Statistical Analysis

Statistical analysis was performed using GraphPad Prism 5.01 for Windows (San Diego, CA, USA) via a repeated-measures one-way analysis of variance (ANOVA) followed by the Bonferroni’s multiple comparison test or two-way ANOVA followed by Bonferroni’s post-tests for evaluating the effects of dose, time, and dose by time on PWL and motor block effects. The dependent variable for these analyses was time in minutes. The Mann-Whitney test as nonparametric analysis and paired *t* test were applied to compare the histopathology scores between time points. The statistical significance was determined as *P*< 0.05. All data were presented as means ± standard error of mean (SEM).

## Results

### Duration of sensory nerve block

**Fig 1:**
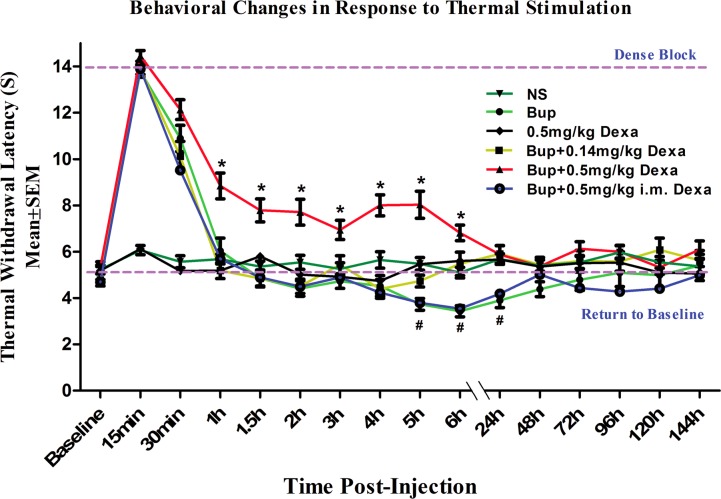
Paw withdrawal latency behavioral changes in response to thermal stimulation after sciatic nerve block with various medications. * indicates P<0.05 when compared with other groups. # indicates P<0.05 when compared with baseline control. The data were reported as mean ± SEM. NS, normal saline; Bup, bupivacaine; Dexa, dexamethasone; i.m., intramuscularly.

Fig 1 shows that high-dose dexamethasone (0.5mg/kg) added to bupivacaine perineurally significantly increased the pain threshold to thermal stimulus up to 360 minutes post-injection when compared with control normal saline, plain bupivacaine, low-dose perineural dexamethasone (0.14mg/kg) with bupivacaine, and plain bupivacaine with intramuscular dexamethasone (0.5mg/kg) (* *P*<0.05). The plain bupivacaine with or without low-dose perineural dexamethasone only produced a short duration of sensory nerve block (less than 60 minutes), while no changes in nociceptive responses were observed in control normal saline and dexamethasone only (0.5 mg/kg) groups.

Perineural dexamethasone extended sensory blockade of the sciatic nerve. To test whether the effect of dexamethasone was via a systemic or perineural mechanism, dexamethasone was administrated intramuscularly, while bupivacaine was deposited around sciatic nerve. The results indicated no effects with the systemic dexamethasone (Fig 1), which suggested the mechanism of action for dexamethasone was at least primarily perineural. In addition, systemic dexamethasone did not nullify the rebound hyperalgesia found with perineural bupivacaine treatment (Fig 1).

### Rebound hyperalgesia after the resolution of bupivacaine nerve block

Interestingly, from 300 minutes to 24 hours after perineural injection, the plain bupivacaine groups with or without intramuscular dexamethasone showed an elevated response to thermal stimulation (rebound hyperalgesia) compared to baseline control groups (*P*<0.05). Treatment groups with either high or low dose perineural dexamethasone in addition to bupivacaine did not show evidence of hypersensitivities to thermal stimulation during our study observation period (Fig 1).

### Duration of motor nerve block

**Fig 2:**
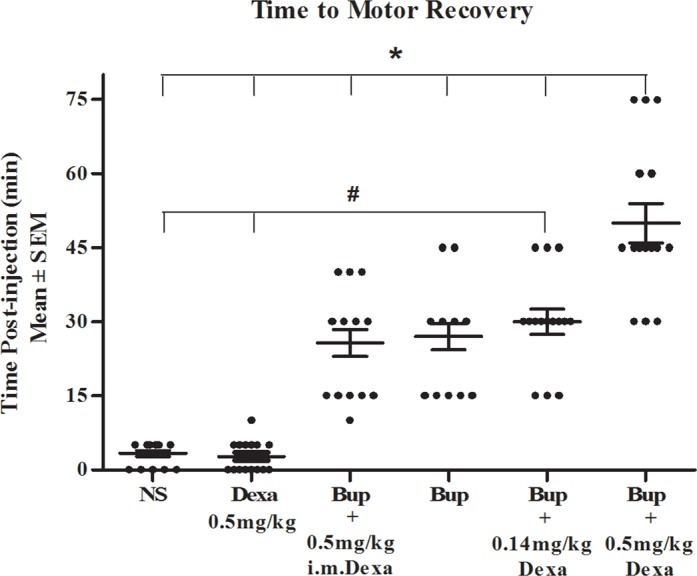
Motor recovery of mouse sciatic nerve block with various medication. * indicates P<0.05 when compared with other groups. # indicates P<0.05 when compared with baseline control. The data were reported as mean ± SEM. NS, normal saline; Bup, bupivacaine; Dexa, dexamethasone; i.m., intramuscularly.

Fig 2 summarizes effects of bupivacaine in the sciatic nerve motor block. Supplemental low-dose perineural dexamethasone (0.14mg/kg) in addition to bupivacaine produced similar motor blockade response compared to bupivacaine with or without intramuscular dexamethasone, while all bupivacaine treated mice had a significantly increased time to motor recovery compared to normal saline or dexamethasone treated mice (*P*<0.05). Interestingly, the combination of high-dose perineural dexamethasone (0.5mg/kg) with bupivacaine significantly prolonged sciatic nerve motor block duration when compared to all other groups (Fig 2, *P*<0.05).

### Histomorphological changes after bupivacaine injection

**Fig 3:**
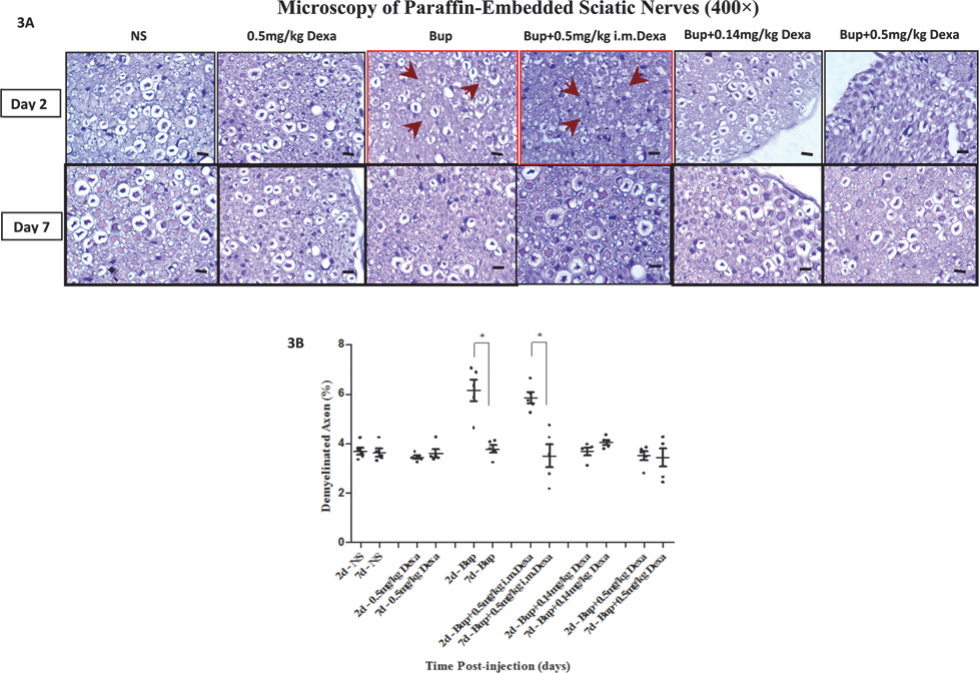
Microscopic changes of sciatic nerves after sciatic nerve block. 3A. istomorphological changes of paraffin-embedded sciatic nerves on day 2 and day 7 after injection (400×, toluidine blue staining). Nerves appeared normal on day 2 and day 7 after injection in most groups, while nerves in the bupivacaine treated group (red box) showed fiber degeneration with vague myelin sheath appearance (arrows) at 2 days after injection; 3B. Percentage of demyelinated axons in sections (n = 5 per group). The percentage of unmyelinated axons in bupivacaine group was significant higher on post-injection day 2 than on day 7 (*P<0.05), while no significant difference was observed in other groups. NS, normal saline; Bup, bupivacaine; Dexa, dexamethasone. Sale bar is at 10μm.

Most axons had a uniform shape and light blue axoplasm ranged from small to large in diameter. Bupivacaine with or without intramuscular dexamethasone injected mouse sciatic nerve showed evidence of mild nerve fiber degeneration with the appearance of a vague myelin sheath (red box) on post-injection day 2 (Fig 3A). The percentage of demyelinated axon was significantly higher on day 2 as compared to day 7 in the plain bupivacaine injection group (6.15±0.43% vs 3.78±0.16%, *P*<0.05), while there was no significant difference among other groups between day 2 and day 7 (Fig 3B, S1 Table).

### Sciatic nerve tissue apoptosis among treatment groups

**Fig 4:**
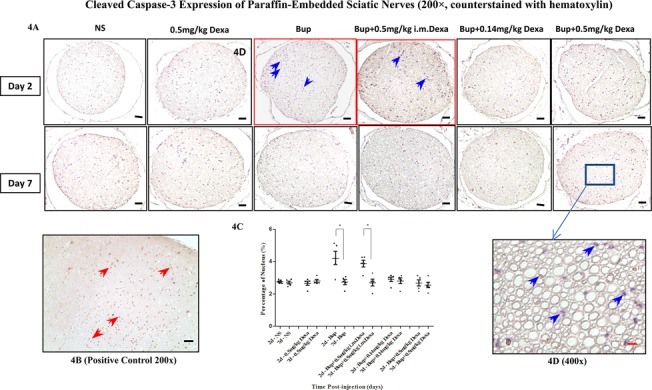
Caspase-3 expression in sciatic nerves. 4A. Immunohistochemical assays for cleaved caspase-3 staining (200×, nucleus counterstained with hematoxylin). There was no significant caspase expression in sciatic nerve axoplasm in all groups; 4B. Caspase-3 expression in positive control tissue (red arrows) (hippocampal neurons, 200×); 4C. Percentage of nucleus in sciatic nerves. The percentage of nucleus staining (blue arrows) in sciatic nerve was significant higher in bupivacaine treated mice on day 2 than day 7 (red box, *P<0.05), while no significant difference was found in other groups. 4D. Caspase-3 expression in mouse sciatic nerve after bupivacaine and high-dose dexamethasone block (400×). NS, normal saline; Bup, bupivacaine; Dexa, dexamethasone. Scale bar: 45 μm (black color),10 μm (red color).

The extent of sciatic nerve injury was further evaluated via the immunoreactivity of cleaved caspase-3. Sciatic nerve axoplasm was negatively stained with caspase-3 in all treatment groups on post-injection day 2 and day 7 (Fig 4). The percentage of nucleus stained in dark blue in the mouse sciatic nerve tissue after plain bupivacaine treatment was significantly higher on day 2 (red box, *P*<0.05) (Fig 4D, S2 Table), which implies that an early proliferative response to injury in Schwann cells happened during Wallerian degeneration [16, 17].

### Schwann cell degeneration after bupivacaine injection

**Fig 5:**
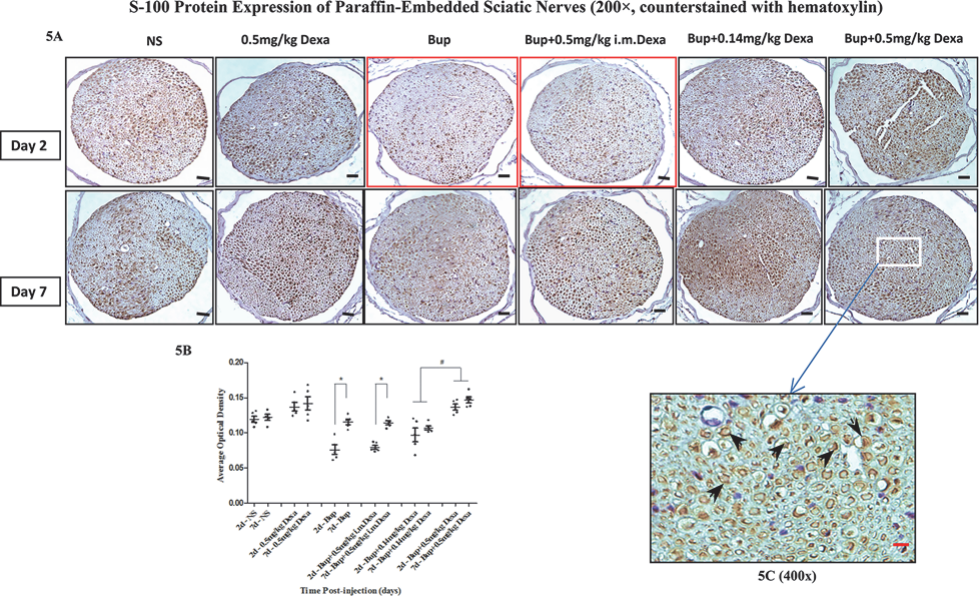
S-100 expression in sciatic nerves. 5A. S-100 protein immunostaining in sciatic nerves (200×); 5B. Immunoreactivity of S-100 expression in different treatment groups on day 2 and day 7. Bupivacaine with or without intramuscular dexamethasone treatment group showed significantly lower S-100 expression on day 2 (red box) than that on day 7 (*P<0.05), while S-100 was expressed significantly higher when treated with perineural bupivacaine and high-dose dexamethasone than treated with perineural bupivacaine and low-dose of dexamethasone (^#^ *P*<0.05); 5C. Immunohistochemical assay for S-100 protein staining (deep brown, black arrows) in each group was primarily localized in the myelin sheath (400×, counterstained with hematoxylin). NS, normal saline; Bup, bupivacaine; Dexa, dexamethasone. Scale bar: 45 μm (black color),10 μm (red color).

In Schwann cells, S100 expression may be related to axon diameter and degree of myelination [18]. Plain bupivacaine treatment on mouse sciatic nerve led to decreased S-100 protein immunoreactivity on post-injection day 2 as compared to day 7 (*P*<0.05, Fig 5A, S3 Table). While the combination dexamethasone and bupivacaine treatment groups did not show a significant difference in S-100 protein expression between day 2 and day 7 within each treatment condition, high dose dexamethasone-treated mouse sciatic nerve appeared to have much higher expression of S-100 protein compared to the low dose dexamethasone treated mice (*P*<0.05, Fig 5B). The S-100 protein was expressed prominently in the cytoplasm of Schwann cells surrounding the myelinated fibers (brown color) as opposed to demyelinated axons (Fig 5C).

## Discussion

To our knowledge, this is the first *in vivo* animal study indicating that dexamethasone added to bupivacaine significantly prolongs the duration of thermal antinociceptive responses in a dose-dependent manner. Dexamethasone also prevents bupivacaine-induced rebound hyperalgesia. These effects of dexamethasone related to perineural mechanism may be related to the prevention of bupivacaine-induced Schwann cell degeneration.

### Prolongation of bupivacaine blockade by high dose perineural dexamethasone

Two groups of investigators have recently reported that intravenous dexamethasone could provide a supplemental analgesia effect similar to the perineurally delivered dexamethasone [10, 11]. However, close examination of their data shows some differences. Fredrickson et al studied effects of dexamethasone in sciatic nerve block and ankle block, and reported that perineural dexamethasone treatment demonstrated an improved analgesia profile at 24 hours, while only minor analgesia benefit by 48 hours, as compared to intravenous dexamethasone treatment [10]. Rahangdale *et al* compared the effects of perineural versus intravenous dexamethasone on sciatic nerve blockade outcomes. Their results show that the perineural dexamethasone group had better pain relief on post-operative day 1, both at rest and with activity [11]. Conversely, another recent clinical report concludes that perineural, but *not* the intravenous administration of dexamethasone 4mg significantly prolongs the duration of effective postoperative analgesia [19]. The effective route of dexamethasone administration is still unclear [20].

The doses of bupivacaine and dexamethasone used in our experiment are equivalent to those used in the clinical setting. Our study shows that the duration of both sensory and motor blockade of bupivacaine is significantly prolonged by high-dose perineural dexamethasone when compared with the other treatment groups. The low-dose perineural dexamethasone did not present any significant prolongation of either sensory or motor block. In addition, dexamethasone’s synergistic effect on bupivacaine also appeared via local, but not systemic mechanism. The intramuscularly injected dexamethasone failed to show any additional effect on the sensory and motor response of perineural bupivacaine. Although the precise mechanism by which dexamethasone exerts its synergistic effect is still unclear, previous studies have speculated that it may be mediated, at least in part, by vasoconstriction and direct action on C-fiber glucocorticoid receptors in peripheral nerves [21, 22]. Therefore, future studies, such as selective glucocorticoid receptor targeting might provide insights on its mechanism [23].

### Bupivacaine Induced Rebound Hyperalgesia

Recently, Williams *et al*. described ‘‘rebound pain” or worsening pain following the resolution of either perineural single injection or continuous infusion of local anesthetics (0.25% levobupivacaine) in humans who had received femoral nerve blocks [24]. Patients commonly describe an intense burning pain initially as the nerve block resolves, which is an undesirable clinical ramification of peripheral nerve blocks [24]. Similarly, 0.5% ropivacaine could also induce a transient episode of rebound heat hyperalgesia following the resolution of sensory blockade in a rat sciatic nerve block model even when no painful surgery was performed in the sciatic nerve distribution [25, 26]. This interesting neurobehavioral phenomenon was also observed in our study. A short period of rebound hyperalgesia to heat stimuli was produced in the 0.5% bupivacaine-only treated mice at time points from 5 to 24 hours after perineural sciatic nerve injection. The appearance of this phenomenon at different time periods may be related to different local anesthetics being used and the study species. Despite the fact that this phenomenon is short-lived, we believe it has clinical significance.

“Rebound pain” after resolution of PNB is generally considered to be due to the mechanical-surgical pain from unopposed nociceptive input that is uncovered after the resolution of the PNB. [25]. The etiology of this “rebound pain” is unknown, but the perineural injection of neurotoxic anesthetics on heat-specific pain fibers may be a possible mechanism for rebound pain [27]. Bupivacaine up-regulation of cyclooxygenase 2 gene expression and resultant increased prostaglandin E2 production at the surgical site has been shown to occur, and contributes to the phenomenon of rebound pain after effects of the LA have dissipated [28]. Therefore, increasing nerve block duration in combination with a multimodal analgesic strategy could decrease the incidence of “rebound pain” after a nerve block resolves.

### Short-term neurotoxicity of bupivacaine and Schwann cells degeneration

Local anesthetics have been associated with an increased inflammatory response, altered nerve permeability, and myotoxicity [29]. Bupivacaine could induce apoptosis in a dose-dependent manner via disrupting mitochondrial membrane and activating caspase 3 and other byproducts [30]. Our results show that bupivacaine administered within our study concentrations did not induce significant apoptosis during the study period. Clinical concentrations of local anesthetics can also alter perineural permeability, produce changes in the endoneural environment, and cause axonal dystrophy [31]. Subperineurial edema following perineural administration of LA suggests that this barrier system is disrupted by LA contributing to nerve fiber injury [32, 33]. It is also possible that subclinical nerve injury in the setting of PNB is quite common and the injury is only clinically significant with the co-existence of other contributing patient-related and procedure-related risk factors. In contrast with the results of a previous study [34], our study showed that the sciatic nerve had more fiber disintegration with vague myelin sheath appearance on day 2 after perineural bupivacaine injection. Interestingly, this was recovered by post-injection day 7.

Schwann cells are myelin sheath-forming cells in the peripheral nervous system involved in many important aspects of peripheral nerve biology, including the nerve development, regeneration, and the maintenance of healthy axons [35, 36]. Axon degeneration is a prominent early feature of most neurodegenerative disorders and can also be induced directly by nerve injury in a process known as Wallerian degeneration, which is a process leading to the removal and recycling of axonal and myelin-derived fragments [37]. During this process, Schwann cells undergo dedifferentiation, proliferation (especially non-myelinating Schwann cells), and recruitment of macrophages into nerve. In the late regeneration phase, non-myelinating Schwann cells transform into myelinating cells [38]. S-100 is preferentially distributed in myelin-forming Schwann cells and may be related to axon diameter and degree of myelination [18]. The expression of S100 could serve as a biomarker for the myelinating Schwann cell, and thus represent the regeneration stage [38, 39]. In our present study, despite lack of caspase-dependent apoptosis evidence in the Schwann cell, the abnormal rebound hyperalgesia behavior, higher percentage of nucleus, and decreased S-100 protein expression on day 2 after plain bupivacaine injection implied the early phase of Schwann cells response. This result suggests that the neurotoxicity of perineural bupivacaine is likely related to its Wallerian degeneration and demyelination, a characteristic change in peripheral nerve degeneration. Although there are observed differences in neuron markers, the small incidences of fiber degeneration and Schwann cell response are of concern. Future studies are indicated to confirm and address the significance of such finding.

### Anti-nociceptive and anti-neurotoxic effects of perineural dexamethasone

A recent *ex vivo* study of the effects of clonidine, buprenorphine, dexamethasone, and midazolam on rat sensory neurons found that a supratherapeutic concentration of each of these four agents alone was far less neurotoxic than a therapeutic concentration of ropivacaine alone at 24 hours [40]. Dexamethasone did not present a synergistic toxic effect with ropivacaine in their *ex vivo* study, therefore offering a modicum of support for the “off-label” safe use of these other additives [40]. On the other hand, steroids may have a direct neurotoxic effect on the peripheral nerve tissue only when the drugs are injected intrafascicularly into the nerve bundle [41]. Our study observed that dexamethasone, when added to bupivacaine could inhibit the occurrence of bupivacaine-induced short-term rebound hyperalgesia both at low and high doses perineurally. Neurotoxicity of plain bupivacaine identified via histomorphology and S-100 expression patterns was no longer significant with the co-administration of preservative-free dexamethasone perineurally. It is possible that perineural dexamethasone could prevent the transient neurotoxicity of bupivacaine and guard against demyelination and Schwann cell degeneration, and therefore demonstrates anti-nociceptive and anti-neurotoxic effects in this mouse sciatic nerve block model.

## Conclusions

In conclusion, perineural, but not systemic administration of dexamethasone, when added to clinical concentration of bupivacaine could not only prolong the duration of sensory and motor blockade of sciatic nerve, but also prevent the bupivacaine-induced reversible neurotoxicity and short-term rebound hyperalgesia after the resolution of block. The protective effect of dexamethasone is likely Schwann cell related.

## Supporting Information

S1 Table Summary statistic of microscopic changes of sciatic nerves after sciatic nerve block. (DOCX)

S2 Table Summary statistic of sciatic nerve injury via immunoreactivity of cleaved caspase-3. (DOCX)

S3 Table Summary statistic of sciatic nerve immunoreactivity of S-100 protein. (DOCX)
