# Supplementary material for: Dexamethasone as Adjuvant to Bupivacaine Prolongs the Duration of Thermal Antinociception and Prevents Bupivacaine-Induced Rebound Hyperalgesia via Regional Mechanism in a Mouse Sciatic Nerve Block Model
Source: PLoS One. 2015 Apr 9;10(4):e0123459. doi: 10.1371/journal.pone.0123459 (PMC4391940; doi:10.1371/journal.pone.0123459)
Supplement: S1 Table — (DOCX) [file pone.0123459.s001.docx]

| **Table S1. Summary statistic of microscopic changes of sciatic nerves after sciatic nerve block** | | | |
| --- | --- | --- | --- |
|  | **Mean ± S.E.M** | |  |
| **Groups** | **Day 2** | **Day 7** | ***P* value** |
| Normal Saline | 3.69 ± 0.15 | 3.63 ± 0.16 | 0.841 |
| 0.5mg/kg Dexamethasone | 3.45 ± 0.07 | 3.61 ± 0.17 | 0.600 |
| Bupivacaine | 6.15 ± 0.43 | 3.78 ± 0.16 | 0.008 |
| Bupivacaine +0.5mg/kg i.m. Dexamethasone | 5.85 ± 0.23 | 3.50 ± 0.47 | 0.008 |
| Bupivacaine + 0.14mg/kg Dexamethasone | 3.68 ± 0.148 | 4.04 ± 0.10 | 0.056 |
| Bupivacaine + 0.5mg/kg Dexamethasone | 3.51 ± 0.19 | 3.42 ± 0.37 | 0.840 |

“Mean” represented percentage of positive changes in each total field of vision (200x magnification), which was quantified with ImageJ 1.49a software.
