# Supplementary material for: Dexamethasone as Adjuvant to Bupivacaine Prolongs the Duration of Thermal Antinociception and Prevents Bupivacaine-Induced Rebound Hyperalgesia via Regional Mechanism in a Mouse Sciatic Nerve Block Model
Source: PLoS One. 2015 Apr 9;10(4):e0123459. doi: 10.1371/journal.pone.0123459 (PMC4391940; doi:10.1371/journal.pone.0123459)
Supplement: S2 Table — (DOCX) [file pone.0123459.s002.docx]

| **Table S2. Summary statistic of sciatic nerve injury via immunoreactivity of cleaved caspase-3** | | | |
| --- | --- | --- | --- |
|  | **Mean ± S.E.M** | |  |
| **Groups** | **Day 2** | **Day 7** | ***P* value** |
| Normal Saline | 2.76 ± 0.05 | 2.71 ± 0.09 | 0.841 |
| 0.5mg/kg Dexamethasone | 2.67 ± 0.13 | 2.80 ± 0.09 | 0.990 |
| Bupivacaine | 4.22 ± 0.41 | 2.74 ± 0.16 | 0.032 |
| Bupivacaine +0.5mg/kg i.m. Dexamethasone | 3.88 ± 0.21 | 2.70 ± 0.21 | 0.016 |
| Bupivacaine + 0.14mg/kg Dexamethasone | 2.90 ± 0.14 | 2.81 ± 0.17 | 0.548 |
| Bupivacaine + 0.5mg/kg Dexamethasone | 2.67 ± 0.18 | 2.55 ± 0.17 | 0.753 |

“Mean” represented percentage of positive changes in each total field of vision (200x magnification), which was quantified with ImageJ 1.49a software.
