# Supplementary material for: Dexamethasone as Adjuvant to Bupivacaine Prolongs the Duration of Thermal Antinociception and Prevents Bupivacaine-Induced Rebound Hyperalgesia via Regional Mechanism in a Mouse Sciatic Nerve Block Model
Source: PLoS One. 2015 Apr 9;10(4):e0123459. doi: 10.1371/journal.pone.0123459 (PMC4391940; doi:10.1371/journal.pone.0123459)
Supplement: S3 Table — (DOCX) [file pone.0123459.s003.docx]

| **Table S3. Summary statistic of sciatic nerve immunoreactivity of S-100 protein** | | | |
| --- | --- | --- | --- |
|  | **Mean ± S.E.M** | |  |
| **Groups** | **Day 2** | **Day 7** | **P value** |
| Normal Saline | 0.119 ± 0.004 | 0.122 ± 0.004 | 0.599 |
| 0.5mg/kg Dexamethasone | 0.137 ± 0.006 | 0.142 ± 0.009 | 0.461 |
| Bupivacaine | 0.076 ± 0.007 | 0.116 ± 0.004 | 0.014 |
| Bupivacaine+0.5mg/kg i.m. Dexamethasone | 0.079 ± 0.003 | 0.114 ± 0.003 | 0.001 |
| Bupivacaine + 0.14mg/kg Dexamethasone | 0.094 ± 0.009 | 0.107 ± 0.003 | 0.339 |
| Bupivacaine + 0.5mg/kg Dexamethasone | 0.137 ± 0.004 | 0.147 ± 0.005 | 0.209 |

“Mean” represented percentage of positive changes in each total field of vision (200x magnification), which was quantified with ImageJ 1.49a software.
